# New rural pension scheme, intergenerational interaction and rural family human capital investments

**DOI:** 10.3389/fpubh.2023.1272069

**Published:** 2023-11-14

**Authors:** Lujie Fan, Jing Hua

**Affiliations:** School of Economics and Management, Ningxia University, Yinchuan, China

**Keywords:** new rural pension scheme, spillover effect, intergenerational interaction, rural family, human capital investment

## Abstract

**Introduction:**

The new rural pension scheme (NRPS) can improve the quality of life for rural older adult individuals; however, can it have a spillover effect on rural household human capital investments through intergenerational interactions?

**Methods:**

Based on data from the China Family Panel Studies (CFPS) in 2010, 2012, 2014, 2016, and 2018 and from the perspective of intergenerational interactions, the spillover effect and influencing mechanism of the new rural insurance policy on rural household human capital investments are empirically tested.

**Results:**

The results show that the participation of families in the new rural insurance policy can significantly promote the human capital investments of rural families, and they are robust. Moreover, the spillover effect of this new policy is significantly different due to the gender, insurance phase, and family income of the insured. Through intergenerational interactions, the new rural insurance policy has an impact on the human capital investments of rural families from the material level of intergenerational economic support, housework and childcare for children and the nonmaterial level of old-age care cognition.

**Discussion:**

Therefore, continuing to promote the coverage of the new rural insurance policy and scientifically improving rural social security through publicity and education to promote benign intergenerational family interactions can improve the accumulation of human capital in rural areas.

## 1. Introduction

Human capital is an important driver of rural revitalization, among which education is the most important channel for human capital formation and accumulation (1). Education is of great significance for improving individuals’ abilities, increasing family welfare and promoting rural economic growth. The 20th report of the Communist Party of China also clearly proposed building a high-quality education system, accelerating the high-quality development of compulsory education and optimizing the allocation of regional educational resources. Faced with the current disparity between urban and rural public education investments and resource allocations in China, optimizing rural family human capital investments is said to be an important issue for some time to come. At the same time, given the transformation of China’s demographic structure, the life expectancy of older adult individuals has been extended, and the modern family structure of three or even four generations exists generally, aggravating the burden on family pensions. In this context, to ensure the basic livelihood of older adult rural residents, the State Council issued the Guiding Opinions on Carrying out the Pilot Project of the new rural social pension insurance policy in 2009 and started its pilot implementation. The pilot covers rural residents who do not participate in urban employee endowment insurance. The new rural pension scheme was officially implemented nationwide in 2012 (2). On the one hand, the implementation of a new rural pension insurance program can increase the disposable income of older adult individuals, reduce future uncertainty, and improve living conditions. On the other hand, as the basic unit of individual life, the family has an obvious two-way feedback and intergenerational relationship. New rural pension insurance programs have a certain spillover effect on the family and even society while satisfying the needs of older adult individuals themselves. In addition, due to the dual influence of traditional Chinese family ethics and the reality of childcare security, family intergenerational interaction behaviour and intergenerational caregiving have become common phenomena in Chinese society, especially in poor areas. Such caregiving implies two-way intergenerational economic support, caregiving support and emotional support, which contribute to the formation of adhesive intergenerational relationships among family members (3, 4). Then, due to intergenerational reciprocity behaviour and altruistic motivations, rural older adult individuals tend to choose the reciprocal family equilibrium relationship of give-and-reward to encourage their children to take care of them. In this way, while improving the welfare of older adult individuals, will the new rural pension insurance program have a spillover effect and thus impact family intergenerational interaction behaviour? We explore the impact of the implementation of a new rural pension scheme on the human capital investments of rural households from the perspective of intergenerational interactions, verify the spillover effect of this scheme at the empirical level using data from China Family Panel Studies (CFPS) in 2010, 2012, 2014, 2016, and 2018, and engage in an in-depth examination of the long-term impact of this scheme on households’ human capital investments. We explore the impact mechanism of intergenerational interaction at the material and immaterial levels to better understand the relationship between the new rural pension scheme and the human capital investments of rural households, improve the old-age security system under demographic changes, and make better use of intergenerational family interactions to optimize the allocation of family resources.

## 2. Literature review

Human capital is an important driving force for high-quality economic development, and the factors affecting the level of human capital investments have been the focus of scholars at home and abroad. In previous studies, the factors affecting household human capital investments mainly include household input from a micro perspective and government public input from a macro perspective (5). At the household level, household wealth, such as household income level, housing wealth and land endowment, and household personality characteristics household characteristics, such as education expectations, family size, parents’ education level and household demographic structure, are the main influencing factors of household human capital investments.

At the microhousehold level, the probability and intensity of household human capital investments are closely related to family wealth status (6, 7). It has basically become a social consensus that income inequality leads to educational inequality (8). Housing wealth has a significantly positive impact on family investments in education (9). Land transfers can increase families’ attention to children’s education and the nonagricultural employment of rural residents, thus increasing family investments in human capital and ultimately promoting the accumulation of the human capital of rural teenagers (10). In addition to family wealth, factors such as increased educational expectations, family size, and parents’ educational level affect the level and probability of family education expenditures (11). Mothers’ ambitions and education level are related to children’s education expenditures to a certain extent, and ambition is a channel for intergenerational mobility (12, 13). Family size is associated with family human capital (14). Regarding the family demographic structure, grandparents affect the educational process of offspring through the specific role they assume in the family (15). There is a crowding-out effect of both the burden of old-age care and the burden of child support on household human capital investments (16). At the macro social level, government support for education is also a major determinant of household investments in human capital (17). But there are three forms of correlations – substitution, complementarity, and no statistical correlation – between public education financial investments and household education expenditures, and the findings of the current study are still controversial (18).

Among the above factors affecting household human capital investments, household wealth is a major influencing factor, and pension insurance is currently an important source of income for older adult individuals in households (19). The new rural insurance, as the main endowment insurance in rural areas, has been implemented since the pilot implementation of the new rural pension scheme in 2009. Some scholars have started to study the effect of the policy. The existing policy evaluation has not only focused on older adult individuals who receive the new rural pension scheme but has also extensively studied the spillover effect of the policy. On the one hand, improvements in the welfare of older adult individuals by the new rural pension scheme is mainly reflected in health status, economic income, mental health and other aspects (20). In the context of universal health insurance, pensions can encourage low-income people to use outpatient and inpatient services and older adult individuals to use preventive health care to obtain timely treatment and promote public health (21, 22). Regarding socioeconomic status, improvements in social welfare is conducive to enhancing the financial satisfaction and quality of life of older adult individuals (23). From the perspective of mental health, increasing the pension can improve the mental health problems caused by depressive symptoms and depression by improving confidence in the future (24). On the other hand, there are also certain spillover effects of the new rural pension scheme in the areas of labour supply, land, and children’s health. The scheme can increase the labour supply of offspring, accelerate the transformation of the rural labour population structure, push more efficient production factors into the market (2), promote the increase in labour supply of groups with high labour supply, and inhibit the labour supply of groups with low labour supply (25). Moreover, while improving the welfare of older adult individuals and reducing their demand for agricultural labour, the scheme weakens the function of land security and accelerates land circulation (26). In this process, the new rural pension scheme plays the role of a catalyst for transforming the rural economic development model (27). In addition, the Chinese family, as a basic living unit, redistributes resources within the family, which can increase the transfer payments and care of older adult individuals to their grandchildren and improve children’s health through the influence of intergenerational interactions (28). Social security payments as a source of exogenous changes in family income have an impact on children’s enrolment (29, 30).

In summary, scholars at home and abroad have studied the influencing factors of household human capital investments and the multidimensional effects of new rural pension schemes. However, most of the literature has focused on the impact of the policy on land, the health of older adult individuals, labour supply, children’s health and other family aspects, while studies related to the microwelfare of household human capital with education as the main feature are still limited. In long-term interdependent family systems, older adult individuals and grandchildren of many families live together. In this context, the relationship between the new rural pension scheme and family human capital investments is an important research topic for current family and even social development. There is a lack of research in the literature on the human capital investments of rural households under the new rural pension scheme, and the underlying mechanisms of intergenerational interactions have not been explored in depth. Compared with the literature, the main contributions of this paper are as follows. (1) Combined with the particularity of China’s family culture and the ageing of society, we set control variables from different dimensions, such as household head and family characteristics, study the impact of the new rural pension scheme on rural family human capital investments, and deeply analyse the spillover effect of the policy. (2) We provide a comprehensive analysis of the differential impact of the policy on household educational inputs in terms of gender of the household head, stage of participation and economic status. (3) From the innovative perspective of intergenerational interactions, we analyse at a deeper level the impact mechanism of the new rural pension scheme on family human capital investments from the material level of care inputs and intergenerational economic support and the nonmaterial level of old age and education cognition to provide a reference for making decisions that promote human capital investments of rural families in China.

## 3. Research hypothesis and analytical framework

The problems of an ageing population and a shortage of young labourers in rural areas are becoming increasingly prominent. In the context of China’s comprehensive implementation of rural revitalization, human capital accumulation is increasingly important. Investments in education, as an effective means of human capital appreciation, is of great significance to the future economic growth of rural areas. At present, rural education resources are relatively scarce compared to those of urban areas; therefore, in addition to focusing on the impact of public education investments on human capital, we can also pay attention to the spillover effect of noneducation policies on human capital investments. It has been shown that the new rural pension scheme not only enhances the well-being of the rural older adult themselves but also has an impact on household education expenditures, children’s health, land transfers, children’s labour time and so on.

In many developing countries, pensions, as fiscal transfer payments with the nature of welfare expenditures, are often independent of people’s incomes. Pensions undoubtedly provide stable, exogenous incomes for many families (31), which helps enhance households’ wealth levels. In rural areas, pensions are important incomes of households, especially for older individuals with limited sources of income. A higher income level is conducive to improving living conditions, enriching spiritual life and promoting physical and mental health (32). In addition, the economic level is an important dimension to measure the family’s socioeconomic status, and the extra income from a pension improves the family’s economic level. The higher the socioeconomic status of the family is, the more it invests in the education of the next generation, and the more it is conducive to the accumulation of human capital for family education. Therefore, the new rural pension scheme not only contributes to the direct improvement of individual welfare status but also changes the family’s educational decision and enhances the total family investment in children’s education in terms of indirect effects (33). Based on the above analysis, the following hypothesis is proposed:

*H1:* The new rural pension scheme has a significant positive impact on the human capital investment of rural households.

According to relevant studies, intergenerational interaction is a collective term for positive behaviours, such as communication, resource sharing, and mutual support among multiple generations possessing kinship, including intergenerational mutual assistance and exchange of economic, goods, and labour services, as well as the comprehension of different generations regarding lifestyle and values (34). We divide intergenerational interaction behaviour into two parts: intergenerational economic support and life care at the material level and old-age cognition and education cognition at the nonmaterial level.

Intergenerational interactions at the material level, such as family financial support and life care, have become normal in rural areas of China. Family pensions through intergenerational economic support originally represented the traditional filial piety ethics in rural areas. However, with the ageing of China’s population, intergenerational transfer payments to support parents have become a serious economic burden for only children. Given the continuous improvements in the social security system, the burden, risk and uncertainty of family pensions are gradually weakening, and the underwriting protection of pension insurance is an important substitute for family pensions. The social pension security mechanism represented by the new rural pension scheme has become an important alternative resource for rural family pensions and allows parents to receive public transfer payments and improve their living economic situations, thus reducing the burden of children’s pensions and family economic burden (35). To a certain extent, this mechanism can also increase the care of older adult individuals for their children’s families and children and ease the pressure on young adults’ lives. In addition, when the wealth level of older adult individuals increases, due to the reciprocity of family members, more intergenerational support of children comes from life care, irregular visits and spiritual comfort, alleviating older adult individuals’ anxiety caused by emotional losses and pension problems. Given the reduction in family pension transfer payments, family decision makers make rational decisions to support older adult individuals and invest in education under certain budget constraints. Moreover, benign intergenerational interactions are conducive to the internal flow of resources so that the spillover effect can be brought into play. These interactions alleviate a certain percentage of household pension expenditures and increase the share of investments in education for the care of young children (36). Based on this, the following hypothesis is proposed:

*H2:* New rural pension schemes facilitate the internal flow of resources through benign intergenerational economic support and care inputs at the material level, which benefit families’ investments in children’s education.

**Figure 1:**
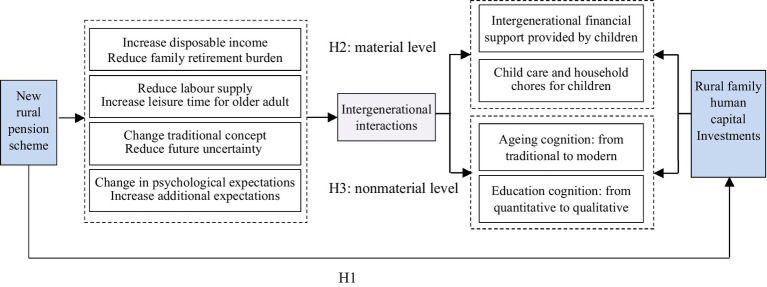
The effect mechanism of the new rural pension scheme on household human capital investments.

In rural China, the idea of passing on the family line and raising children for old age is deeply rooted; therefore, the intergenerational interaction of family care is common in the family unit. In recent years, the establishment of and improvements in the social security system have been continuously deepened, and some direct or indirect economic incentives have had a certain impact on intergenerational interactions and traditional concepts. Not only does it have a substitute effect on family pensions but also it is a change in the concept of raising children and passing on the family. The implementation of the new rural pension scheme has alleviated the worries of older adult individuals in terms of pensions and has a better vision of their pension expectations. Therefore, Given the basic satisfaction of material conditions, older adult individuals are more likely to pursue nonmaterial emotional needs, which may generate additional expectations for the next generation, such as expectations for the future development of their children (37). Compared with the current pursuit of the number of children in rural areas, many families have started to pay more attention to the quality of their offspring given an increase in social public welfare. Based on the above analysis, we propose the following hypothesis (see Figure 1):

*H3:* New rural pension schemes change traditional pension cognition, raise the importance of education, and further improve the family human capital investment level.

## 4. Methods

### 4.1. Data sources

In this paper, data from the China Family Panel Studies (CFPS) organized and implemented by the China Social Science Survey Center of Peking University are used as the dataset for the empirical research, covering the basic information of 16,000 households and individuals in 25 provinces/municipalities/autonomous regions. This dataset reflects social, economic, demographic, educational, and health changes in China. The database provides comprehensive and detailed data for the empirical study. In order to safeguard the basic livelihood of the older adult, ease the pressure on families to support them, and solve the problem of “providing for the older adult,” China issued the Guiding Opinions on the Pilot Project of New Rural Pension Scheme on 1 September 2009, exploring the establishment of a New Rural Pension Insurance system that combines individual contributions, collective subsidies and government subsidies. The policy was piloted in 10 percent of the country’s counties in 2009, expanded to 23 percent of the counties in 2010, and fully rolled out in 2012. Within the scope of the pilot programme, people aged 60 and over who are not covered by urban basic pension insurance and who have a rural household registration do not have to pay and can receive a monthly pension. Rural residents between the ages of 16 and 59 (excluding schoolchildren) who are not covered by urban basic pension insurance can participate in the NRPS by making contributions for a cumulative total of 15 years, and receive a monthly pension when they reach the age of 60. Those who have less than 15 years away from pensionable age can also receive pension by making annual contributions or supplementary contributions. Pensions consist of a basic pension and a personal account pension. The nationally determined basic pension rate was 55 yuan per person per month in 2009, increasing to 70 yuan in 2012, 88 yuan in 2014, and 93 yuan in 2020. Individual account pensions are paid on a monthly basis, at a rate of 139 per person, divided by the total amount stored in the individual account.

**Figure 2:**
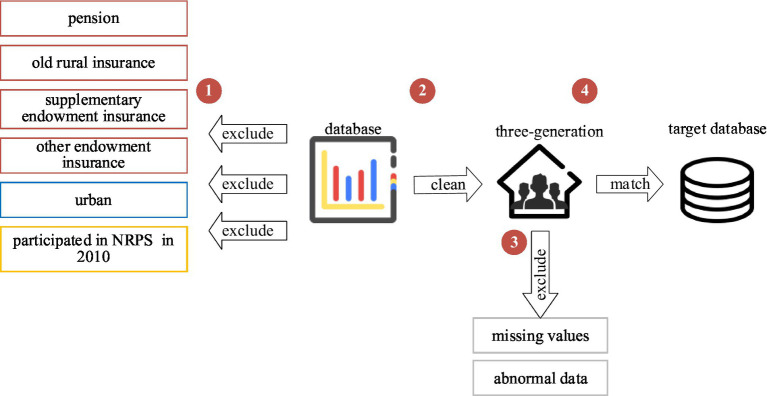
Schematic of sample selection.

We use panel data synthesised from 2010, 2012, 2014, 2016, and 2018. To accurately measure the impact of the new rural pension scheme on family human capital investments, we first exclude individuals and families who receive a pension and have old rural insurance, supplementary endowment insurance and other endowment insurance in the data. After that, according to the participation rules of the new rural pension insurance program, samples with an urban household registration are eliminated. And to study the spillover effects of the full coverage of the new rural pension insurance program in 2012, the sample that had participated in the insurance in 2010 was excluded. Next, the study sample was restricted to rural households with three or more generations to ensure that these households had both older adult individuals who might receive new rural pension insurance and children who were in school. Finally, the database was matched to remove missing values and abnormal data, resulting in a valid sample of 10,540. In addition, due to the missing intermediate mechanism variables required in the three years of data from 2012, 2014, and 2016, the paper uses two periods of data from CFPS 2010 and 2018 to discuss the intrinsic mechanism of action between the new rural pension insurance program and rural family human capital investments (see Figure 2).

### 4.2. Variable selection and descriptive statistics

#### 4.2.1. Explained variable

In this paper, we use total family education expenditures to measure the explained variable family human capital investment. This is captured by the household question, “How much did your family spend on education in the past 12 months?” (38).

#### 4.2.2. Core explanatory variables

In this paper, the core explanatory variable is the insurance participation behaviour of rural households. Specifically, households with individuals participating in or receiving a new rural pension scheme are regarded as the individual’s family to participate in pension insurance. The responses to the question of “participation items” were selected, the number of households choosing the new rural pension scheme was set to 1, and the rest of the choices were set to 0. To prevent the sample results from being influenced by other insurance items, we only take the sample of farmers who have participated in new rural pension insurance programs and those who have not participated in any pension insurance program. It should be noted that in the CFPS questionnaire in 2010, there is no option for a new rural pension scheme as an endowment insurance project. Therefore, we use the response to “when did you join new rural pension scheme” in the 2012 personal database to judge whether the 2010 sample participated in the program (39).

#### 4.2.3. Intermediate mechanism variables

To test the mechanism of the impact of the new rural pension scheme on the human capital investments of rural households from the perspective of intergenerational interactions, based on the previous analysis and combined with data availability, we use intergenerational interaction as an intermediate mechanism variable. Specifically, care input and intergenerational economic support at the material level. as well as old-age cognition and education cognition at the nonmaterial level, are selected as intergenerational interactions for the mechanism test.

#### 4.2.4. Control variables

Combining data availability and following the principle of exogeneity, control variables were selected at the individual and family levels, including household size, household *per capita* income, and household savings rate at the household level and gender, years of education, marriage, and health status of the participants at the individual level.

The descriptive statistical results of the main variables are shown in Table 1.

**Table 1:** Description of main variables and descriptive statistical analysis.

Year		2010		2012		2014		2016		2018	
Variable	Variable description	Mean	SD	Mean	SD	Mean	SD	Mean	SD	Mean	SD
Explained variable											
Total education expenditure	Total family educational expenses (in Chinese yuan), logarithmically	4.673	3.759	4.695	3.827	4.933	3.903	5.543	3.849	5.744	3.841
*Per capita* education expenditure	Average educational expenses per child (in Chinese yuan), logarithmically	4.288	3.493	4.398	3.617	4.629	3.691	5.203	3.646	5.378	3.626
Explanatory variable											
Household participation	Household participating in new rural pension scheme is set to 1 and 0 otherwise	0	0	0.658	0.474	0.816	0.388	0.632	0.482	0.611	0.488
Head of household participation	Household head participating in the new rural pension scheme is set to 1 and 0 otherwise	0	0	0.532	0.499	0.691	0.462	0.478	0.5	0.459	0.498
Household head characteristics											
Gender	Household head is set to 1 for male and 0 for female	0.836	0.370	0.626	0.484	0.570	0.495	0.555	0.497	0.540	0.499
Marriage	Household head has a spouse set to 1 and no spouse set to 0	1	0	0.929	0.257	0.924	0.265	0.913	0.282	0.904	0.294
Education level	Household head years of education	6.566	3.814	5.805	4.417	6.077	4.073	6.277	4.087	6.636	4.072
Health	Physical condition of household head, 1 → 5 unhealthy → healthy	4.178	1.029	2.797	1.227	2.974	1.269	2.886	1.253	2.869	1.262
Work	Set to 1 if household head is employed, set to 0 if no employment or retirement	0.673	0.469	0.864	0.343	0.856	0.351	0.831	0.375	0.829	0.376
Household characteristics											
Filial piety	Whether children are responsible for caring for their parents when they are sick	0.127	0.332	0.123	0.328	0.137	0.344	0.167	0.373	0.144	0.351
Family Size	Total number of family members	5.495	0.948	5.177	0.912	5.215	0.929	5.190	0.944	5.251	0.929
Household *per capita* income	Household’s income *per capita* (in Chinese yuan)	4575.925	4393.318	7999.669	7890.458	8898.464	7774.319	11354.676	9185.234	12720.736	9971.503
Household savings rate	Total household savings as a proportion of income	0.183	0.967	1.973	15.595	1.028	16.694	0.607	9.338	0.476	2.014
Household health care expenditure	Household expenditures on health care (in Chinese yuan) taken as a logarithm	6.324	2.509	7.043	2.036	7.088	2.383	7.218	2.413	7.232	2.484
Intergenerational interaction											
Care inputs	Older adult person helps with household chores or childcare	0.056	0.229	0.086	0.281	—	—	0.196	0.397	0.201	0.401
Intergenerational financial support	Children provide financial support to older adult individuals	0.033	0.178	0.070	0.255	—	—	0.174	0.379	0.202	0.402
Perceptions of Ageing	Household head’s perception of heirlooms	4.168	1.064	—	—	3.759	1.410	—	—	4.305	1.013
Education awareness	Household head’s perception of future children’s success	4.639	0.675	—	—	4.122	1.060	—	—	4.594	0.756

Table 1 shows that the current participation rate in the new rural pension scheme is relatively low, and there is room for the further release of the popularity of pension insurance. Family expenditure on education is rising year by year, which shows that families are attaching greater importance to it. Regarding household demographic structure, most rural families have five or six members and there are relatively more families living with the older adult and children. Regarding family background, the majority of the household heads in rural areas are male, and the health status of the household heads is generally good and job opportunities are also increasing. However, their educational background is generally poor, indicating that the existing educational level of residents in rural areas is still low and that it is necessary to study human capital investments in rural households to facilitate the understanding of the importance of families, government and society to children’s education and improve such education.

### 4.3. Model setting

The paper focuses on the impact of the new rural pension scheme on the human capital investments of rural households. To validate H1, we use a two-way fixed effects model for time and individuals as follows:


(1) $${\text{lnedu}}_{\text{it}}=\beta_{0}+\beta_{1}{\text{Nrps}}_{\text{it}}+\beta_{2}X_{\text{it}}{+u}_{i}{+v}_{t}+\varepsilon_{\text{it}}$$

In Equation (1), $lnedu_{it}$ represents the logarithmic value of household education expenditures, and $Nrps_{it}$ represents whether rural households participate in the new rural pension scheme. The variable is a dummy variable and takes the value of 1 if participating and 0 otherwise. $X_{it}$ and $\beta_{2}$ represent a series of control variables, such as household level, head of household level and their corresponding regression coefficients.$\;\beta_{0}$ and $\beta_{1}$ are parameters to be estimated, and $\beta_{1}$ is the coefficient of focus in this paper, reflecting the impact of the new rural pension scheme on the human capital investment of rural households. $\;\beta_{0}$ is the constant term, $u_{i}$ and $v_{t}$ represent household individual fixed effects and time fixed effects. $\varepsilon_{it}$ is the random error incorporating both individual and time dimensions. This model is used in the basic regression and heterogeneity analysis sections.

According to the previous analysis, the new rural pension scheme mainly affects family human capital investments through the paths of care inputs, intergenerational economic support, education cognition and pension cognition. Referring to the practice of Chen et al. (40) and Peng et al. (41), we further explore the impact mechanism of the new rural pension insurance program on the human capital of rural households, and the specific model is as follows:


(2) $$M_{\text{it}}=\gamma_{0}+\gamma_{1}{\text{Nrps}}_{\text{it}}+\mu X_{\text{it}}+\varepsilon_{\text{it}}$$

In the formula, $M_{it}$ is the intergenerational interaction of the intermediate mechanism variable, $Nrps_{it}$ is the core explanatory variable of the new rural pension scheme, $X_{it}$ represents a series of control variables that affect household human capital investments and insurance participation behaviour, and $\varepsilon_{it}$ is a random disturbance term. To test the mechanism of care input, intergenerational economic support at the material level and education cognition and old-age cognition at the nonmaterial level, we adopt the logit and ordered probit models for the estimation, respectively.

## 5. Empirical analysis

### 5.1. The effect of the NRPS on the human capital investment of rural households

#### 5.1.1. Baseline regression

To explore the impact of the new rural pension scheme on household human capital investments, we use total education expenditure of rural households as the explanatory variable. The fixed-effects regression results of the panel data are detailed in Table 2. Models (1–1), (1–2), and (1–3) successively add a series of control variables. In all regression results, the coefficients of the new rural pension scheme participation variable are positive and significant at the 1% level, indicating that participation in the new rural pension scheme helps improve human capital investments of rural households. Hypothesis 1 is verified. Models (1–3) show that household participation in the new rural pension scheme increases household human capital investments at a rate of 39.9%. Therefore, it can be concluded that the participation of rural households in the new rural pension scheme has a significantly positive effect on total household education expenditures, which also indicates that this new scheme has a spillover effect. In the regression results of the control variables, the work of the household head variable is significant at the 1% level, and the coefficient is positive, indicating that household education expenditure is higher when the head of household has a job, probably because the household head has a higher family income if he has a job, and has a certain status in social life, so he will pay more attention to the welfare of the family and pay more attention to the quality of education. A larger household size increases household education expenditures, probably because there are more children in the family, so the education expenditure is larger.

**Table 2:** Influence of the new rural pension scheme on rural family human capital investment.

Explained variable	Education		
	(1–1)	(1–2)	(1–3)
Explanatory variable			
Household participation	0.434*** (0.131)	0.431*** (0.131)	0.399*** (0.129)
Household head characteristics			
Gender	—	−6.788 (4.289)	−5.813 (4.225)
Marriage	—	−0.303 (0.454)	−0.881* (0.451)
Education level	—	0.0002 (0.060)	0.008 (0.060)
Health	—	0.081 (0.054)	0.086 (0.053)
Work	—	0.496*** (0.166)	0.529*** (0.163)
Household characteristics			
Filial piety	—	—	0.063 (0.165)
Family size	—	—	0.915*** (0.086)
Household *per capita* income	—	—	0. 0000125 (0.0000076)
Household savings rate	—	—	−0.002 (0.007)
Household health care expenditure	—	—	0.055** (0.024)
_cons	4.501*** (0.099)	8.445*** (2.786)	2.876 (2.791)
Family fixed effects	Yes	Yes	Yes
Year fixed effects	Yes	Yes	Yes
*R*^2^	0.0479	0.0516	0.0816
Number of Obs.	10,540	10,540	10,540

***, **, and * indicate significance at the 1%, 5%, and 10% levels, respectively; standard deviations are in parentheses (same below).

#### 5.1.2. Robustness test

In this section, we replace the explanatory variables, change the explanatory variables, and increase the study sample to conduct the robustness test. The regression results are shown in Table 3.

**Table 3:** Robustness test results.

	Education		
	(2–1)	(2–2)	(2–3)
Household participation	0.393*** (0.123)	—	0.380*** (0.120)
Head of household participation	—	0.261** (0.123)	—
Household head characteristics	Yes	Yes	Yes
Household characteristics	Yes	Yes	Yes
_cons	4.016 (2.663)	3.032 (2.794)	3.094 (2.858)
Family fixed effects	Yes	Yes	Yes
Year fixed effects	Yes	Yes	Yes
*R* ^2^	0.0700	0.0803	0.0805
Number of Obs.	10,540	10,540	11,190

Considering that the number of children in different families will have an impact on the overall education expenditure, this part of the total education expenditure of the explanatory variables will be replaced by the “average education expenditure of the child” for the robustness test. The results are shown in Model (2–1) in Table 3. The new rural pension scheme has a significantly positive impact on the human capital investment of rural households, and the regression results obtained do not shift substantially and the previous results remain robust.

Considering the differences between the insurance participation behaviour of households and individuals, to further test the robustness of the above results, we use the insurance participation behaviour of household heads as the core explanatory variable to verify the basic regression results according to the literature. The results for Model (2–2) are presented in Table 3 and show that the participation behaviour of household heads is still significantly and positively related to household education expenditures, indicating that the receipt of pensions by household heads can significantly promote household human capital investments. These results are similar to the benchmark regression results, further indicating their robustness.

Because of the existence of pilot areas in 2010, this paper uses the panel data synthesised by excluding households participating in NRPS in 2010. This part extends to all insured households by re-adding the excluded samples in the regression. Models (2–3) show that the implementation of the NRPS has a significantly positive impact on household human capital investment at the 1 percent level, consistent with the results of the benchmark regression.

#### 5.1.3. Endogeneity problem

To better address the endogenous problems caused by missing variables and possible reverse causality between the new rural pension scheme and household human capital investments, we refer to Zheng et al. (42) and Zhou et al. (43), whether the local county was a NRPS pilot at the survey year is defined as an instrumental variable to eliminate the estimation errors caused by self-selection bias. However, since the new rural pension program was implemented nationwide at the end of 2012, and the policy has achieved full coverage, and the survey areas after 2012 are all pilot counties for the new rural pension program. Given the data reasons, the instrumental variables in this paper for 2014, 2016, and 2018 are invariant and the time fixed effects cannot be controlled, so the instrumental variable method is not used in this paper to test the endogeneity problem. We use a multi-temporal policy effect estimation methodology.

Although the two-way fixed effects model controls for the variables that affect household human capital investments at the household and individual levels, on the one hand, there may be other unobservable variables may simultaneously affect participation in the new rural pension scheme and household education expenditures, resulting in missing variable bias. On the other hand, two-way fixed effects models usually contain only two groups (treatment and control) and two periods (before and after policy treatment), but with heterogeneous treatment effects, many studies have more than two treatment time points, where earlier treated samples become the control group for the later treated samples and the negative weighting problem may make the estimated coefficients biased, and so it leads to implausible empirical estimates. To alleviate the problem of estimation bias, we first refer to the idea of Callaway and Sant’Anna (44). Estimation is carried out using the CSDID model, which basically consists of avoiding the use of individuals who have already been treated as the “bad control group” and selecting only the “good control group” to estimate the group-period average treatment effect, and then weighted average on both group and period dimensions to obtain the average treatment effect of the policy. At the same time, the use of two-way fixed effects models to estimate the dynamic effects of policies may also lead to the coefficients for each period becoming difficult to interpret because of cross-contamination across periods. Based on this consideration, the DID_M_ estimator proposed by de Chaisemartin and D’Haultfoeuille is used to deal with the problem (45).

**Figure 3:**
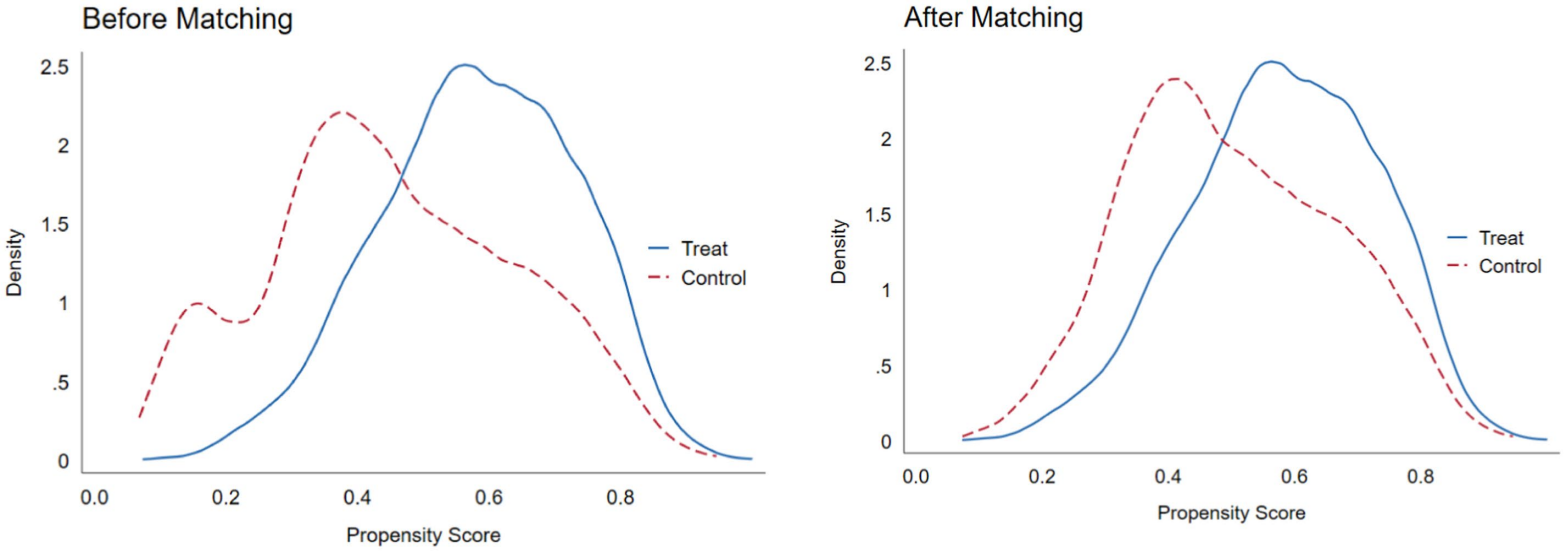
Nuclear density map of PSM matching.

CSDID is used to mitigate the problem of estimation bias, while the average treatment effect of the new rural pension scheme and the average effect of all groups in different years are examined, and the results are shown in Table 4. The policy effects in model (3–1) are all significantly positive at the 1% level, which indicates that the policy has a facilitating effect on the human capital investment of rural households. The results of model (3–2) show a slight increase in the DID_M_ estimates compared to the base regression results, but the change is relatively small. This suggests that the base model bias issue may be present, but does not materially affect the results. And PSM-DID_M_ is used to avoid potential selectivity bias. It obtained the propensity score value by logit regression of the control variables through the dummy variable of whether or not to participate in the new rural pension scheme, and used k-nearest neighbour matching to determine the weights. The mean value of the matched treatment group is 5.38 and the mean value of the control group is 5.00, which is closer. ATT after matching was 0.372, which suggests that the NRPS does significantly increase the level of investment in the human capital of rural households. To ensure that the PSM results meet the “conditional independence assumption,” it is necessary to test the balance of matching variables between the treatment group and the control group, requiring that there is no significant systematic difference between the samples. The absolute values of the standardised deviations of the matched covariates are less than 10% and most of the t-test results do not reject the original hypothesis that there is no difference between the treatment group and the control group, which passes the balance test, thus justifying the use of PSM-DID_M_. Meanwhile, it draws the density function plot of the propensity score value to test the matching effect of the treatment and control groups. As shown in Figure 3, the probability densities of the propensity score values are close to each other after matching, indicating that the matching effect is better. So the feasibility and reasonableness of the PSM-DID_M_ are further proved on the basis of the common support domain. Table 4 models (3–3) reveal the DID_M_ regression results after using propensity score for k-nearest neighbour matching. Their coefficient is higher than those of the estimated results of the benchmark regression and is still positive at the 5% significance. This finding shows that the positive impact of the new rural pension scheme on the human capital investment of rural households is still verified after controlling for the endogeneity problem of sample selection bias. So the conclusions all suggest that the heterogeneity treatment effect has a more limited impact on the estimation results in this paper.

**Table 4:** Endogeneity tests: treatment effects.

Variable	Education		
	(3–1) CSDID	(3–2) DID_M_	(3–3) PSM-DID_M_
ATT	1.244*** (0.361)	—	—
Household participation	—	0.407** (0.185)	0.539** (0.200)
Household head characteristics	Yes	Yes	Yes
Household characteristics	Yes	Yes	Yes
Family fixed effects	Yes	Yes	Yes
Year fixed effects	Yes	Yes	Yes
Number of Obs.	2,620	2,683	2022

Due to space constraints, the logit regression results during PSM matching, the diagram of the common support domain and the balance test results of PSM are not shown in the paper, as shown in the Supplementary material.

#### 5.1.4. Heterogeneity analysis

The paper further divides the samples into groups according to age, gender and household income to explore the differences in the spillover effect of insurance participation on household human capital investments among different groups.

To examine the heterogeneity of the impact of the policy on family education expenditures in different stages of insurance participation, this section classifies the family samples according to the age required by the policy and discusses the impact of the new rural pension insurance program on the human capital investments of households in the insured but uncollected and collected segments. The regression results are detailed in Table 5 and show that the new rural pension scheme significantly increases household human capital investments in receiving stages but has no significant effect on participants under 60 years of age who have not yet received the NRPS. The reason is that the insured under 60 years old can only expect stable pension security in the future, whereas for aged 60 and above, they have been able to directly obtain the pension, which can increase their welfare, satisfy their basic needs, and even improve their sense of well-being and confidence in the future, so they are willing to use it for other purposes. Thus, compared with the group that is insured but does not receive it, the group in the stage of receiving insurance that receives it promotes investment in the human capital of the household. Thus, compared with the group that is insured but does not receive it, the group in the stage of receiving insurance that receives it promotes investment in the human capital of the household.

**Table 5:** Regression results for different ages of enrolees.

	Education			
	(4–1)	(4–2)	(4–3)	(4–4)
	Over 60	Below 60	Over 60	Below 60
Household participation	0.795*** (0.265)	0.276 (0.198)	0.758*** (0.265)	0.204 (0.195)
Household head characteristics	No	No	Yes	Yes
Household characteristics	No	No	Yes	Yes
_cons	4.635*** (0.327)	4.700*** (0.119)	0.957 (1.482)	−2.341* (1.202)
Family fixed effects	Yes	Yes	Yes	Yes
Year fixed effects	Yes	Yes	Yes	Yes
*R* ^2^	0.0260	0.0630	0.0467	0.1142
Number of Obs.	3,185	7,355	3,185	7,355

Gender difference is one of the factors that affects the human capital investments of rural households. New rural pension schemes to increase the level of human capital investments in households may also differ among household heads of different genders. We divide the total sample into female and male subsamples according to the gender of the household head, and the results are shown in Table 6. Female household heads pay more attention to children’s education than do male household heads, and the new rural pension scheme can significantly improve the level of human capital investments of female-headed households. The reason for the above results may be that women are in a weak position in the household under the long-term historical influence of “men farming and women weaving” and “men taking the lead outside the household and women taking the lead inside the household,” and male household heads are predominant. However, with the progressive emphasis on education in society and as the main group of people who take care of children, women are the heads of the family and are more willing than men to invest more of their stable incomes from pension insurance in their children’s education. Doing so represents better nurturing and care for children, gives them access to more educational resources, increases the family’s resistance to future risks, and exchanges human capital accumulation for life security in old age.

**Table 6:** Regression results for different participants’ genders.

	Education			
	(5–1)	(5–2)	(5–3)	(5–4)
	Female	Male	Female	Male
Household participation	0.669 *** (0.206)	0.289* (0.169)	0.602*** (0.204)	0.271 (0.167)
Household head characteristics	No	No	Yes	Yes
Household characteristics	No	No	Yes	Yes
_cons	4.647*** (0.284)	4.497*** (0.102)	0.759 (1.474)	−1.128 (0.950)
Family fixed effects	Yes	Yes	Yes	Yes
Year fixed effects	Yes	Yes	Yes	Yes
*R* ^2^	0.0712	0.0393	0.1037	0.0752
Number of Obs.	3,879	6,661	3,879	6,661

To explore the impact of the new rural pension scheme on family human capital investments in different income classes, family income is divided into low income, middle income and high income according to the quantile of the mean household net income. The regression results are shown in Table 7. The results indicate that for low-income households, participation in the NRPS leads to an increase in household expenditure on education, while for middle-income and high-income households, there is no significant effect. Compared to families with higher income, families with low income usually have a low socio-economic status. The additional income from the pension is likely to improve the overall income level of the family and play a relatively more important role in family expenditures, helping the older adult and the education of their children to a certain extent. Families with higher income may attach relatively higher importance to education itself and spend more on education. This additional income received by the family accounts for a small proportion of the total family income and the likelihood of investing it in childcare costs is relatively low. In general, the difference in income leads to a gap in human capital investments among different families, which further intensifies the differences in human capital endowments among different classes and thus causes larger gaps among families and their incomes.

**Table 7:** Regression results of income disparity.

	Education					
	(6–1)	(6–2)	(6–3)	(6–4)	(6–5)	(6–6)
	Low income	Moderate income	High income	Low income	Moderate income	High income
Household participation	0.661** (0.284)	0.417 (0.348)	0.040 (0.278)	0.616** (0.286)	0.355 (0.348)	0.075 (0.271)
Household head characteristics	No	No	No	Yes	Yes	Yes
Household characteristics	No	No	No	Yes	Yes	Yes
_cons	3.978*** (0.158)	4.009*** (0.261)	4.251*** (0.416)	−0.179 (1.716)	−1.508 (2.140)	−3.173 (1.931)
Family fixed effects	Yes	Yes	Yes	Yes	Yes	Yes
Year fixed effects	Yes	Yes	Yes	Yes	Yes	Yes
*R* ^2^	0.0461	0.0703	0.0855	0.0658	0.1080	0.1477
Number of Obs.	3,512	3,340	3,688	3,512	3,340	3,688

### 5.2. Mechanisms of the impact of the new rural pension scheme on human capital investments in rural households

The family, as an individual living unit, has innate unity and trust. Two-way feedback intergenerational relationships are obvious in traditional society. Under the long-term influence of traditional concepts, most of rural older adult care is based on intergenerational economic support as a means of “family pension.” As an exogenous shock to rural family wealth (46), the new rural pension scheme not only reduces the financial burden of children supporting the older adult to a certain extent, but may even improve the sense of well-being of the older adult, and in turn, the older adult provide their children with livelihood and even financial assistance. And among them, the flow of family resources, the power and status of the older adult in the family, as well as the support of children for older adult individuals are mainly affected by the intergenerational interaction pattern of the family (47). A balanced and orderly benign intergenerational interaction is conducive to the distribution and flow of family resources. Therefore, we analyse the mechanism from both material and immaterial levels to examine whether intergenerational interactions play a role in the impact of the policy on household human capital investments.

Considering the situation of grandparents taking care of children’s household chores and children providing economic support to their parents, the behavioural changes of individuals in the family under the influence of the NRPS are explored at a deeper level to analyse whether intergenerational interactions at the material level play a role in the influence of the policy on the investment in human capital of rural families. The first variable is that grandparents look after children or help with household chores. The current combination of inadequate childcare services, the increase in the number of dual-income families, traditional cultural concepts of succession, and intergenerational family reciprocity has made intergenerational care common in three-generation families. Therefore, after the older adult have secured their basic livelihood, we presume that they may use their leisure time to help their children, so as to fulfill the value of positive family security. The second variable is the financial support provided by children to their parents. As an important part of the social security system for the older adult, the new rural pension scheme will have a substitution effect on supporting for the older adult in the family to a certain extent, which can alleviate the financial burden, reduce some of the upward intergenerational economic support, and improve the distribution of income.

Models (7–1) and (7–2) in Table 8 report the effects of participation in the NRPS on the above mechanism variables at the material level. Model (7–1) shows that participating in the new rural pension scheme reduces the frequency of older adult individuals helping their children do housework and take care of their children. It is possible that the withdrawal of older adult individuals from the labour market to age at home under the impact of the new rural pension scheme. Although a large amount of human capital of the older adult has been accumulated, due to the improvement of their own welfare status and a certain degree of economic independence, the need for their children’s life care and economic help is reduced. As a result, many older adult individuals live more apart from their children, causing them to lack environmental conditions and opportunities to help their children look after their children and do housework. Therefore, it is likely that in the context of the relaxation of the family’s budget constraint, some help is provided to children through intergenerational economic support, giving them more resources to invest in family human capital. As shown in Model (7–2), under the influence of the new rural pension scheme, the financial support provided by the children to their grandparents has decreased. This suggests that the implementation of the policy has improved the wealth level and income expectations of older adult individuals. Thus, children in rural households provide less financial help to their grandparents to some extent, leading to a relative increase in household wealth. Subsequently, the implementation of the policy has not only directly alleviated the pressures of living on older adult individuals themselves but also has indirectly reduced the burden of family care and pensions and has resulted in higher investments of the family income in children’s education.

**Table 8:** Mechanisms of the effect of the new rural pension scheme on family human capital in-vestments.

Intergenerational interaction	Material level		Nonmaterial level	
	(7–1)	(7–2)	(7–3)	(7–4)
	Care inputs	Intergenerational financial support	Perceptions of ageing	Education awareness
Household participation	−0.209* (0.125)	−0.248* (0.135)	0.156*** (0.044)	−0.033 (0.048)
Household head characteristics	Yes	Yes	Yes	Yes
Household characteristics	Yes	Yes	Yes	Yes
_cons	−1.463*** (0.385)	−0.037 (0.399)	—	—
*R* ^2^	0.1166	0.1656	0.0104	0.0036
Number of Obs.	4,547	4,547	4,547	4,547

The next step is to examine how intergenerational interactions at the non-material level play a role in the process of the impact of the NRPS on household human capital investments by considering perceptions of ageing and education awareness. The first variable is household head’s perception of heirlooms. Considering the cultural traditions of succession and family continuity in rural areas, there may be a trade-off between the number and quality of children. Under the influence of a strong idea of succession, it is likely to emphasise the number of children at the expense of investing in the education of each child. The second variable is the importance that the head of the household perception of future children’s success. If household head believes that it is important for children to be successful and attaches importance to the future development, then education, as a key way to improve human capital, is likely to be the choice of the members of the household to invest more resources in their children, so that if their children’s level of wealth increases in the future, they will be more benefited.

As shown in Model (7–3), there is a significant effect of participation in the new rural pension scheme on the perception of family retirement. From the results, it can be seen that participation in the NRPS at the 1 per cent level of significance will increase the importance of the family’s succession, and there is an impact on perceptions of retirement. May be due to the long-term impact of history, the traditional concept of raising children to prevent old age in rural areas still has a deep impact, the policy of “social pension” on the children of “family pension” replacement role is relatively limited, so the participation of the NRPS has not reduced but rather increased its weakening function. In the model (7–4), there is no significant effect on the importance of children’s success. Overall, the new rural pension insurance program has a positive impact on household human capital investments through three paths: increased awareness of grandparents taking care of children’s household chores, children providing financial support, and perceptions of ageing.

## 6. Conclusion

We focus on the human capital investments of rural households in China and discuss the spillover effect of the new rural pension scheme on rural household education investments. Based on data from the China Family Panel Studies (CFPS) in 2010, 2012, 2014, 2016, and 2018, we use a two-way fixed effect model to study the impact of the new rural pension insurance program on household human capital investments and adopts the treatment effect to solve the endogeneity problem.

We find that (1) rural households participating in the new rural pension scheme can significantly increase the level of household human capital investments. (2) The treatment effect model is used to conduct the endogeneity test, it is found that the policy has a positive contribution to household human capital investments, and the results are robust. (3) The spillover effect of the new rural pension insurance program is different depending on the family’s stage of participation, family income and gender of the household head. The spillover effect of the policy is more significant for participants who are female, are in the recipient stage, and are in low-income households. (4) The new rural pension scheme affects household human capital investments mainly through intergenerational interactions in terms of intergenerational financial support for children at the material level, taking care of their children’s household chores, and the cognitive aspects of retirement at the immaterial level. According to the above research conclusions, we propose the following countermeasures.

First, the new rural pension scheme has a positive externality on the education expenditures of rural households in China. We should continue to promote the breadth and depth of the new rural pension scheme, ensure the stable development of the rural social security system, and deepen the pension insurance policy and transfer payment policies such as tax reform in rural areas. Doing so is important to improve the quality of life of rural older adult individuals and investments in the human capital of families. The level of social security in many rural areas is still low, making it necessary to scientifically and reasonably increase the proportion of low-income groups participating in insurance, focus on improving the level of social security for women, strengthen the old-age security function of insurance, improve the psychological expectations of rural residents, and enhance the ability to resist future risks, which are all conducive to promoting family human capital investments and high-quality economic development in rural areas. Second, we can promote benign intergenerational family interactions through publicity and education. We should not only strengthen children’s responsibility of caring for older adult individuals and focus on the intergenerational support of families in noneconomic aspects such as living companionship, psychological health and care for older adult individuals but also help rural households liberate their mindsets, weaken traditional concepts such as passing down the family line and raising children for old age, and strengthen the importance of education to the family. In addition, we can enhance family human capital accumulation through high-quality education investments, improve confidence in children’s future incomes, and reduce future uncertainty. Third, we should accelerate the construction of a high-quality education system, promote the high-quality and balanced development of education, increase public education investments in rural areas, especially remote rural areas and poor mountainous areas, and ensure the equity of education resources. Meanwhile, we should broaden the income channels of rural families and increase family investments in education to promote the healthy development of rural education.

## Data availability statement

The original contributions presented in the study are included in the article/Supplementary material, further inquiries can be directed to the corresponding author.

## Author contributions

LF: Data curation, Formal analysis, Supervision, Validation, Visualization, Writing – original draft, Writing – review & editing. JH: Conceptualization, Formal analysis, Funding acquisition, Methodology, Project administration, Resources, Supervision, Visualization, Writing – original draft, Writing – review & editing.
