# Supplementary material for: New rural pension scheme, intergenerational interaction and rural family human capital investments
Source: Front Public Health. 2023 Nov 14;11:1272069. doi: 10.3389/fpubh.2023.1272069 (PMC10682713; doi:10.3389/fpubh.2023.1272069)
Supplement: Supplementary file 1 [file Table_1.docx]

Supplementary Material

New rural pension scheme, intergenerational interaction and rural family human capital Investments

# Supplementary Figures and Tables

## Supplementary Figures


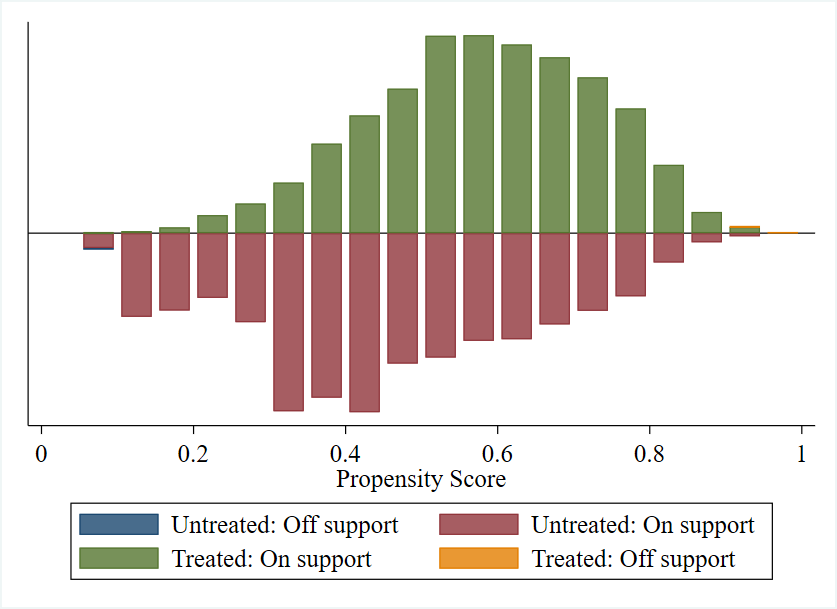


**Supplementary Figure 1.** K-nearest neighbour matching common support domain results

## Supplementary Tables

**Supplementary Table 1.** Logit estimation for propensity score matching

| Variable | Coefficient | Standard errors | Z |
| --- | --- | --- | --- |
| Gender | -0.297 | 0.027 | -10.82 |
| Marriage | -0.092 | 0.054 | -1.68 |
| Education level | 0.012 | 0.003 | 3.65 |
| Health | -0.231 | 0.010 | -22.60 |
| Work | 0.701 | 0.034 | 20.65 |
| Filial piety | -0.052 | 0.038 | -1.36 |
| Family Size | -0.002 | 0.014 | -0.16 |
| Household per capita income | 0.000017 | 0.0000016 | 11.36 |
| Household savings rate | 0.006 | 0.002 | 2.37 |
| Household health care expenditure | 0.033 | 0.005 | 6.18 |
| _cons | 0.041 | 0.099 | 0.42 |
| LR statistics | 1465.19 | | |
| R^2^ | 0.1004 | | |
| Number of Obs. | 10540 | | |

**Supplementary Table 2.** Sample balance test results

| Variable | Treated | Control | Bias（%） | T-value | P-value |
| --- | --- | --- | --- | --- | --- |
| Gender | 0.578 | 0.573 | 1.0 | 0.53 | 0.597 |
| Marriage | 0.930 | 0.916 | 5.8 | 2.79 | 0.005 |
| Education level | 6.357 | 6.274 | 2.0 | 1.05 | 0.295 |
| Health | 2.876 | 2.876 | 0.7 | 0.37 | 0.711 |
| Work | 0.874 | 0.877 | -0.6 | -0.36 | 0.718 |
| Filial piety | 0.142 | 0.144 | -0.4 | -0.20 | 0.843 |
| Family Size | 5.234 | 5.207 | 2.9 | 1.54 | 0.125 |
| Household per capita income | 10277 | 10126 | 1.8 | 0.86 | 0.390 |
| Household savings rate | 0.727 | 0.622 | 1.0 | 0.97 | 0.330 |
| Household health care expenditure | 7.244 | 7.179 | 2.7 | 1.51 | 0.130 |
